# Efficacy and safety of platelet-rich plasma for acute nonarteritic anterior ischemic optic neuropathy: a prospective cohort study

**DOI:** 10.3389/fmed.2024.1344107

**Published:** 2024-03-13

**Authors:** Xin Jin, Junxia Fu, Ruju Lv, Xiaolu Hao, Song Wang, Mingming Sun, Guangcan Xu, Qi Zhang, Lei Zhang, Yan Li, Quangang Xu, Baoke Hou

**Affiliations:** ^1^Senior Department of Ophthalmology, The Third Medical Center of PLA General Hospital, Beijing, China; ^2^Department of Ophthalmology, School of Medicine, Xinhua Hospital Affiliated to Shanghai Jiao Tong University, Shanghai, China; ^3^Department of Ophthalmology, Jinan University, Jinan, China; ^4^Department of Ophthalmology, The Second Affiliated Hospital of Anhui Medical University, Hefei, China

**Keywords:** platelet-rich plasma, nonarteritic anterior ischemic optic neuropathy, prospective controlled trial, OCT angiography, efficacy and safety analyses

## Abstract

**Background:**

As the most common acute optic neuropathy in older patients, nonarteritic anterior ischemic optic neuropathy (NAION) presents with varying degrees of visual acuity loss and visual field defect. However, there is no generally accepted treatment for NAION.

**Objectives:**

To evaluate the efficacy and safety of platelet-rich plasma (PRP) for patients with acute NAION within 2 months.

**Design:**

A prospective, nonrandomized controlled trial.

**Methods:**

Twenty-five eyes of 25 patients were enrolled. Of them, 13 received anisodine hydrobromide and butylphthalide-sodium chloride injection continuously for 10 days as basic treatment in the control group, and 12 received two tenon capsule injections of PRP on a 10 days interval as an additional treatment in the PRP group. We compared the best-corrected visual acuity (BCVA) and capillary perfusion density (CPD) of radial peripapillary capillaries and the moth-eaten eara of the peripapillary superficial capillary plexus and deep capillary plexus at 1 day (D1) before the first PRP treatment and 7 days (D7), 14 days (D14), and 30 days (D30) after the first PRP injection. Ocular and systemic adverse effects were assessed.

**Results:**

In the PRP group, a better BCVA occurred at D30 (adjusted *p* = 0.005, compared with D1, recovered from 0.67 ± 0.59 to 0.43 ± 0.59), and a significant improvement in CPD was observed at D30 (adjusted *p* < 0.001, *p* = 0.027, *p* = 0.027, compared with D1, D7, D14, in sequence, the value was 35.97 ± 4.65, 38.73 ± 4.61, 39.05 ± 5.26, 42.71 ± 4.72, respectively). CPD at D7 in the PRP group was better than that in the control group (*p* = 0.043). However, neither BCVA nor the moth-eaten area index were significantly different (all *p* > 0.5) between the two groups. The main adverse effect was local discomfort resolved within 1 week, and no other systemic adverse events occurred.

**Conclusion:**

Tenon capsule injection of PRP was a safe treatment for AION and could improve capillary perfusion of the optic nerve head and might be helpful in increasing short-term vision in patients with acute NAION.

## Introduction

As the most common acute optic neuropathy in older patients, nonarteritic anterior ischemic optic neuritis (NAION) is characterized by acute or subacute, painless, usually monocular vision loss that progresses over several hours to days and presents with diffuse or segmental optic disc edema often with peripapillary flame-shaped retinal hemorrhages (1, 2). It occurs worldwide with an annual incidence ranging from 2.3 to 10.3 cases per 100,000 persons among adults over age 50 years (1, 3, 4). With visual acuity loss from 20/20 to no light perception (NLP) and a typical arcuate or altitudinal visual field defect particularly in the inferior visual field and a limited improvement (2), a variety of therapies have been attempted, most of which are empirical, such as noninvasive or minimally invasive therapeutic options—oral glucocorticoids or anti-platelet agents, intravitreal injections of triamcinolone or anti-vascular endothelial growth factor (VEGF) agents and hyperbaric oxygen therapy, and surgical solutions—optic nerve decompression. Regrettably, there is no generally accepted treatment for NAION (5–7). To date, the exact cause of ischemia remains uncertain, and the acute hypoperfusion of vascular networks originating from the posterior ciliary arteries is the most widely accepted primary trigger. The main factors—vascular risk factors and a small, crowded optic nerve—contribute to the worsening of optic nerve ischemia (1, 2, 8). Most therapeutic interventions mainly target the possible causes of ischemia, including correction of vascular risk factors, treatment of thrombosis, vasodilation, neuroprotection, and treatment of compartment syndrome (7).

Platelet-rich plasma (PRP) is a preparation of autologous plasma enriched with a higher concentration of platelets, and it possesses high potential for regeneration, which allows for greater release of growth factors and biologically active proteins and then activates the wound-healing cascade by stimulating neoangiogenesis and collagen production (9). PRP preparation involves the collection of a small sample of an individual’s blood and centrifugation to separate platelets from other components (10). Clinically, PRP can be used alone or in conjunction with topical and oral therapies. Due to the ease of use and generally high safety profile, it has propelled itself into mainstream tissue engineering, showing excellent potential for hair restoration, skin rejuvenation, and osteochondral lesions (11–13) and providing a wide range of prospects in ophthalmology from ocular surface—corneal epithelial wound healing such as ulcers, dry eye, and burns (14–18) to ocular fundus—retinitis pigmentosa (19), macular holes (20, 21), and retinal hole repair in recurrent retinal detachment (22).

However, research on the therapeutic effect of PRP on optic nerve diseases is scarce. Our study aimed to evaluate the effectiveness of PRP in improving short-term visual outcomes and the microcirculation of the optic nerve head in acute NAION patients, and the safety of local injection was also assessed.

## Methods

### Study design and participants

We conducted a single-center nonrandomized controlled trial that included all NAION subjects presenting at the Senior Department of Ophthalmology, the Third Medical Center of Chinese People’s Liberation Army General Hospital between February 2022 and August 2022. Our study was in accordance with the tenets of the Helsinki Declaration and the ICH-GCP guidelines, and the study protocol was approved by the Ethics Committee for Human Research of the hospital (approval number KY2021-031). Written informed consent was obtained from all participants.

The diagnosis of acute NAION was primarily based on the clinical diagnosis (1). All patients underwent a comprehensive evaluation before enrollment, including best-corrected visual acuity (BCVA), visual field (when BCVA was more than 0.1), intraocular pressure (IOP), slit-lamp microscope, fundus color photo, optical coherence tomography (OCT), orbital magnetic resonance imaging (MRI) and hematological examination. The inclusion criteria were as follows: (1) age < 75 years, (2) acute and painless loss of vision or visual field defect, (3) optic disc swelling of the affected eye under the direct ophthalmoscope, (4) within 2 months from onset to treatment, and (5) complete follow-up evaluation including 7-, 14-, and 30 days follow-up. The exclusion criteria were as follows: (1) IOP higher than 20 mmHg, (2) concomitant other ophthalmic disorders, including but not limited to corneal opacity, severe cataract, vitreoretinal or other optic nerve diseases, (3) erythrocyte sedimentation rate and C-reactive protein examination suggested arteritic ischemic optic neuropathy, (4) previous history of ocular trauma or ophthalmic surgery, (5) combined with blood system diseases that may affect PRP preparation, and (6) combined with neurological diseases such as neuromyelitis optica spectrum disorders, myelin oligodendrocyte glycoprotein antibody-associated diseases, Alzheimer’s disease, and Parkinson’s disease, which may affect the optic nerve.

All recruited patients were intravenously administered an iodine hydrobromide and butylphthalide-sodium chloride injection for 10 consecutive days as basic treatment. For the PRP group, the first tenon capsule injection of PRP was given on the first day, and the second PRP administration was given on the last day during the basic treatment period as an intensive treatment and without any other additional treatment.

### Visual function examination

All patients underwent a visual function evaluation using a standard logarithmic visual acuity chart at each visit, including 1 day before the first PRP injection (D1) and 7- (D7), 14- (D14), and 30 days (D30) follow-ups after the first PRP injection, and the decimal visual acuity was converted into the logarithm of the minimum angle of resolution (logMAR) scale for statistical analysis.

### Optical coherence tomography angiography imaging and assessment

**Figure 1:**
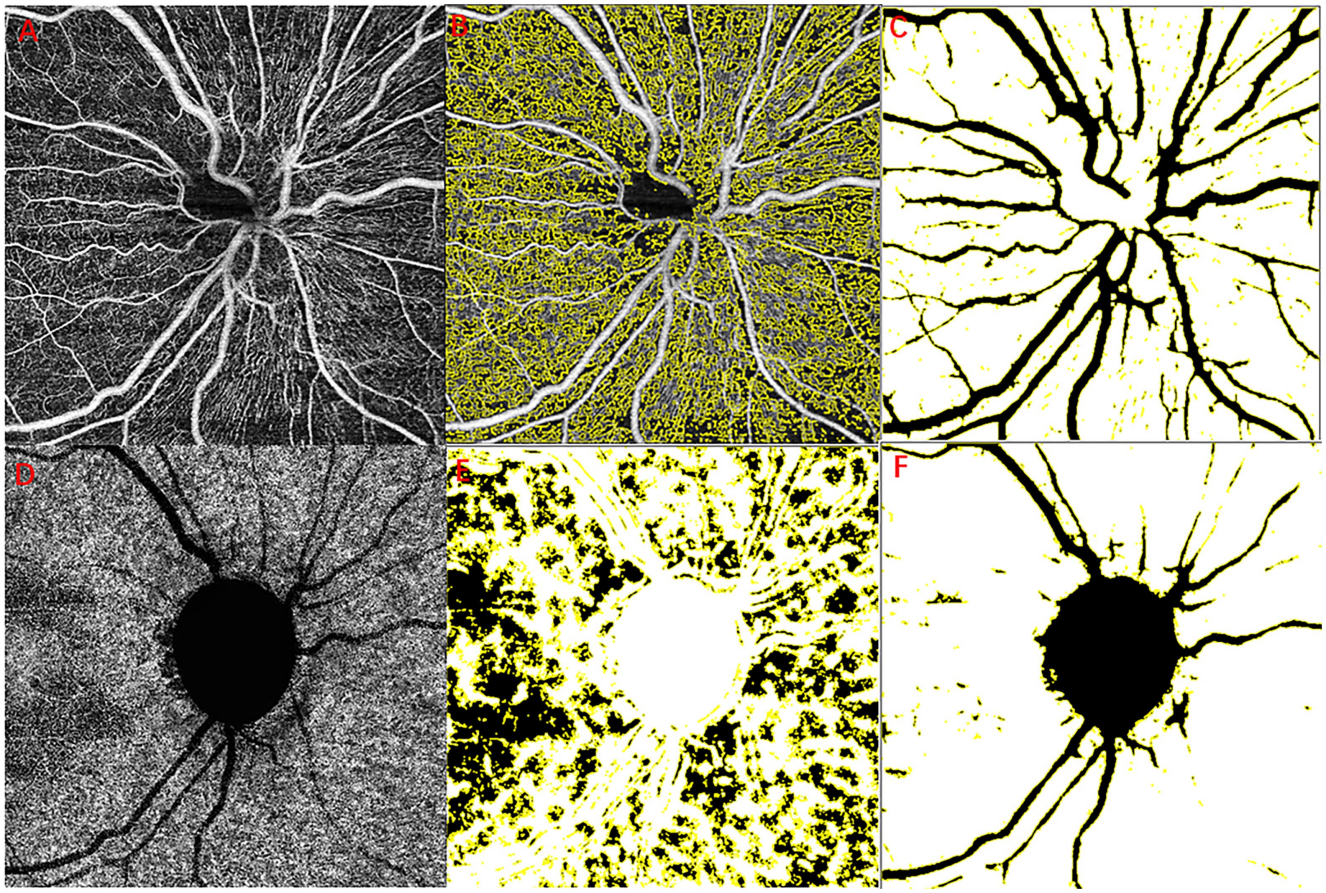
Example images for retinal layer segmentation and large vessel removal. RPC layer blood flow diagram **(A–C)**, original optic disc margin threshold **(A)**, optic perfusion areas **(B)**, and the major branch retinal blood vessels **(C)**. Choroidal capillary blood flow diagram **(D–F)**, original chorioidal capillary layer **(A)**, no-perfusion areas **(E)**, and the major branch retinal blood vessels and optic disc **(F)**. RPC, radial peripapillary capillaries.

All recruited subjects underwent optical coherence tomography angiography (OCTA) scan with a swept-source OCT (YG-100K Yalkaid, TowardPi Medical Technology, Beijing, China) with their eyes dilated and with room lighting turned off. Raster-pattern retinal scans were obtained through the optic nerve head, using scanning patterns of 6 × 6 mm^2^ in all patients. Images with a quality score below 40 or images with obvious motion artifacts or signal loss were excluded. Obvious motion artifacts were defined subjectively as image artifacts seen on the retinal scans, such as a horizontal frame shift larger than the average diameter of retinal vessels or a distorted oval appearance of the optic nerve head. The vascular scanning model of the TowardPi OCTA revealed precise perfusion in four disparate layers: the optic nerve head layer, vitreous layer, radial peripapillary capillaries (RPC), and choroid. Specifically, the optic nerve head layer encompasses the area between the retinal pigment epithelium (RPE) and the inner limiting membrane (ILM). Furthermore, using the TowardPi OCTA, the optic nerve head layer can be further divided into the surface capillary plexus (SCP) and deep capillary plexus (DCP). In our study, the optic nerve head layer and RPC were chosen to assess optic disc blood flow. The graphs were analysed using ImageJ software (1.8.0_112, https://imagej.nih.gov/ij/, National Institutes of Health, Bethesda, Maryland, United States). We calculated the capillary perfusion density (CPD) of RPC as described in a previous study (23) and the moth-eaten area index for the peripapillary SCP and DCP. We selected the optic disc margin threshold (Figure 1A), showing perfusion areas (Figure 1B), and the optic disc region, except for the major branch retinal blood vessels (Figure 1C). The moth-eaten area index was defined as the total weighted area of nonperfused vasculature per unit area in a measurement area by means of marking no-perfusion areas (Figure 1E) and the optic disc region, with the exception of the major branch retinal blood vessels and optic disc (Figure 1F).

### Tenon capsule injection of PRP

**Figure 2:**
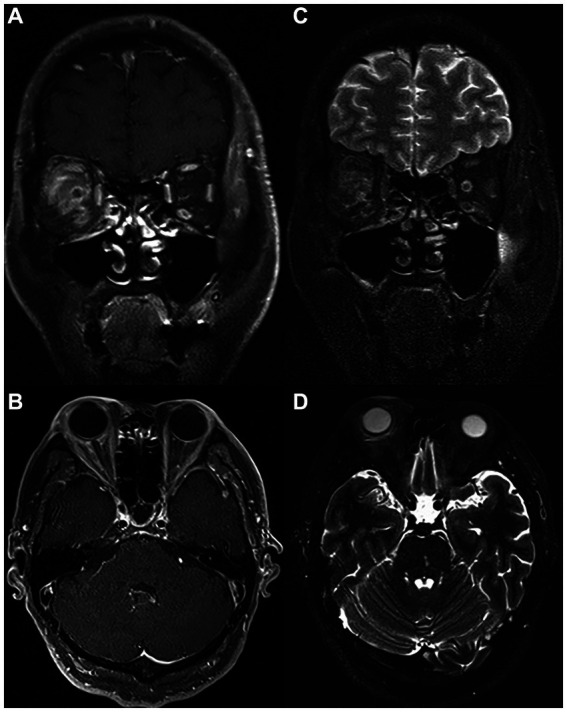
MRI scan of the same patient on the day of PRP injection. Coronal **(A)** and **(B)** axial T1 postcontrast images showing successful injection of PRP in the right Tenon capsule near the inner part of the ball with marked postcontrast enhancement. Coronal **(C)** and **(D)** axial T2-weighted images showing extensive T2 high signal in the fascia tissue behind the ball of the right eye. PRP, platelet-rich plasma; MRI, magnetic resonance imaging.

PRP administration was performed on all case group patients, who received two capsule injections on a 10 days interval during hospitalization. During the preparation session, PRP was obtained by differential centrifugation from approximately 10 mL of autologous blood collected in the operating room. During the treatment session, each patient received 1.5 mL of PRP fluid on the nasal and temporal side of the optic nerve in the Tenon capsule near the inner part of the ball by an experienced ophthalmologist (by BH). As shown in Figure 2, hyperintensity on T2-weighted imaging and enhancement on T1-enhanced scanning were observed after PRP administration, which hinted at successful delivery of the drug to the Tennon capsule.

### Statistical analysis

For patients with simultaneous bilateral eye attack, only one eye was randomly selected using a random number table to avoid selective bias. Statistical analysis was performed with SPSS version 22.0 (IBM Corp., Armonk, NY, United States). Continuous data are presented as the mean ± standard deviation, and categorical variables are expressed as rates and percentages. We used the Mann–Whitney U test to compare the differences for continuous variables and the chi-square test, correct chi-square test, or Fisher’s exact test to compare categorical variables between the two groups. The Friedman test, together with the *post hoc* Wilcoxon signed-rank test, was used to compare BCVA, CPD, and moth-eaten area index at the four time points in each group. Statistical significance in the Wilcoxon signed-rank test was adjusted by Bonferroni correction. We defined *p* < 0.05 as statistically significant.

## Results

### Baseline characteristics of recruited NAION patients

**Figure 3:**
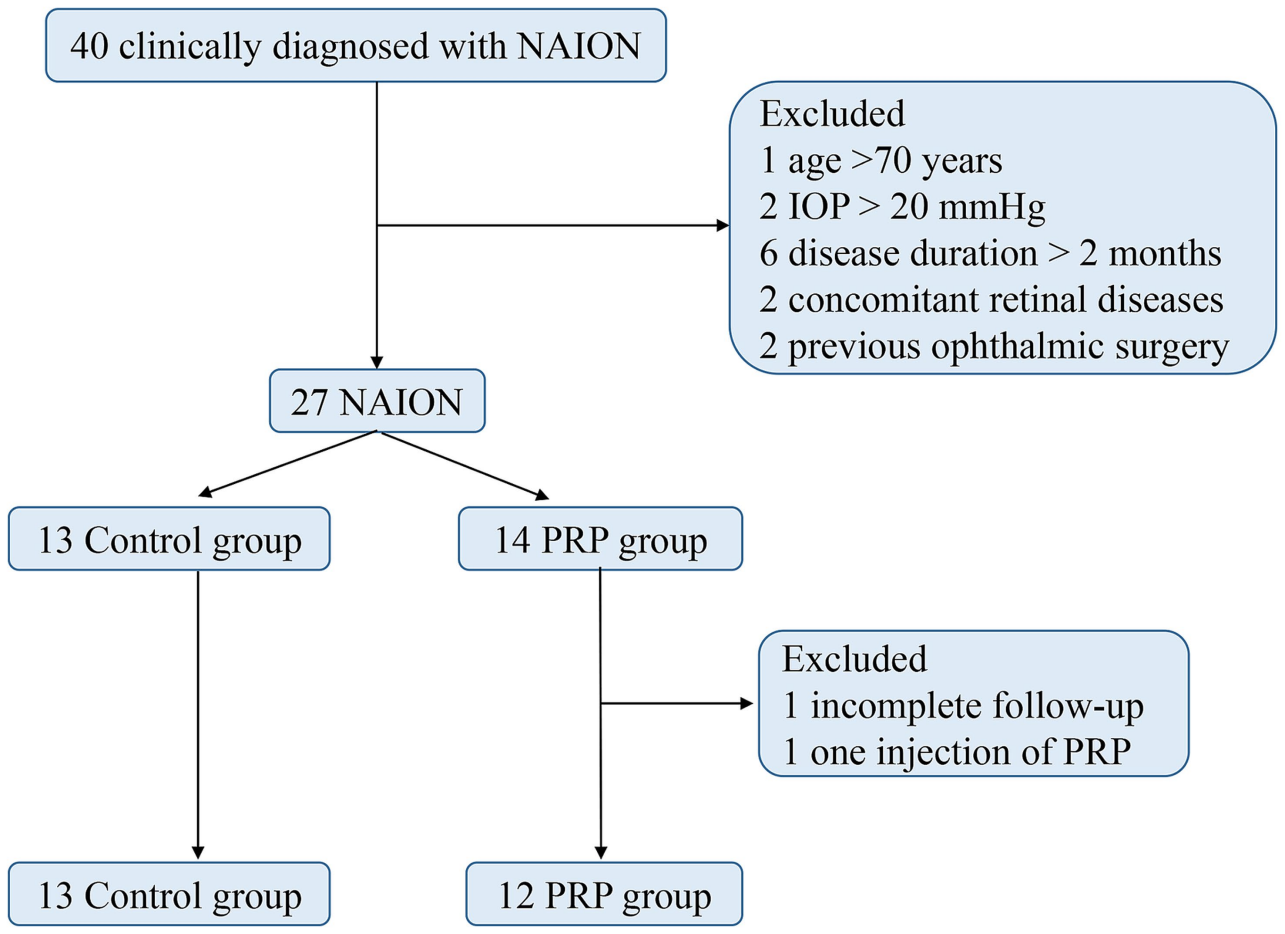
Study flow chart. NAION, nonarteritic anterior ischemic optic neuropathy; PRP, platelet-rich plasma; IOP, intraocular pressure.

The study flow is shown in Figure 3. Finally, a total of 25 consecutive patients, consisting of 13 (52%) in the control group and 12 (48%) in the PRP group, were included in our study. The baseline characteristics of the two groups are shown in Table 1. Among them, 21 (84%) were male, the mean age of onset was 50.84 ± 9.64 years, the mean body mass index (BMI) was 24.56 ± 1.84 kg/m^2^, 24 (96%) were unilaterally involved, 12 (48%) had a history of NAION, and 12 presented a small cup-to-disc ratio or a crowded optic nerve. In terms of the subtypes of visual field defects, 15 presented diffuse defects, 10 presented quadrant defects, and none of them presented central or paracentral scotoma. Compared with the PRP group, a greater proportion of small cup-to-disc ratio or a crowded optic nerve appeared in the control group (*p* = 0.003). No significant differences were found in other clinical characteristics between the two groups (all *p* > 0.05). For the PRP group, the median time from onset to PRP window was 25.5 days.

**Table 1:** Baseline characteristics of the recruited NAION patitents.

	Total	Control group	PRP group	*p*
Study subjects (eyes)	25	13	12	
Male (*n*, %)	21 (84%)	10 (76.9%)	11 (91.7%)	0.647^c^
Age of onset (mean ± SD, years)	50.84 ± 9.64	51.08 ± 11.44	50.58 ± 7.74	0.806^a^
BMI (mean ± SD, kg/m^2^)	24.56 ± 1.84	24.62 ± 1.32	24.48 ± 2.35	0.605^a^
Unilateral eye onset (*n*, %)	24 (96%)	12 (92.3%)	12 (100%)	0.999^d^
History of NAION (*n*, %)	12 (48%)	5 (38.5%)	7 (58.3%)	0.320^b^
Presence of small cup-to-disc ratio or a crowded optic nerve (*n*, %)	12 (48%)	10 (76.9%)	2 (16.7%)	**0.003** ^b^
Type of visual field defect (*n*, %)				
Diffuse defect	15 (60%)	8 (61.5%)	7 (58.3%)	0.999^c^
Quadrant defect	10 (40%)	5 (38.5%)	5 (41.7%)	
Central or paracentral scotoma	0	0	0	
Onset to PRP window (median, IQR, days)	—	—	25.5 (18.8, 37)	—
Systemic diseases				
Hypertension (*n*, %)	11 (44%)	6 (46.2%)	5 (41.7%)	0.821^b^
Diabetes mellitus (*n*, %)	12 (48%)	6 (46.2%)	6 (50%)	0.848^b^
Hyperlipidemia (*n*, %)	10 (40%)	3 (23.1%)	7 (58.3%)	0.165^c^
Hyperhomocysteinemia (*n*, %)	2 (8%)	0	2 (16.7%)	0.220^d^
Nocturnal hypotension (*n*, %)	4 (16%)	3 (23.1%)	1 (8.3%)	0.647^c^
Obstructive sleep apnea (*n*, %)	10 (40%)	6 (46.2%)	4 (33.3%)	0.806^c^
History of ischemic heart disease (*n*, %)	1 (4%)	1 (0.7%)	0	0.999^d^
History of stroke (*n*, %)	0	0	0	—
History of malignant tumor (*n*, %)	0	0	0	—

NAION, nonarteritic anterior ischemic optic neuropathy; PRP, platelet rich plasma; BMI, body mass index; *n*, number; SD, standard deviation; IQR, interquartile range.Bold: *p* < 0.05.

a Mann–Whitney test.

b Chi-square test.

c Correct chi-square test.

d Fisher’s exact test.

As depicted in Table 1, nearly half of the patients had hypertension, diabetes mellitus, hyperlipidemia, and obstructive sleep apnea, one had a history of ischemic heart disease, 2 had hyperhomocysteinaemia, and 4 had nocturnal hypotension overall. None of them had a history of stroke or malignant tumor. There were no significant differences (all *p* > 0.05) in systemic diseases between the two groups.

### Changes in visual function

When comparing the mean logMAR visual acuity at the same time points, no difference was found between the two groups (all *p* > 0.05, Table 2). As expected, no significant difference was found in the mean logMAR VA of the studied eyes at four time points in the control group (Friedman *p* = 0.852, Table 3). In the PRP group, the VA at D1 and D7 ranged from 0.67 ± 0.59 to 0.59 ± 0.56 and did not change significantly after the first PRP treatment (adjusted *p* > 0.999, Table 4). Although no significant change was found after 14 days of follow-up (adjusted *p* = 0.347, Table 4), a better BCVA occurred at the 30 days follow-up than at D1 (adjusted *p* = 0.005, Table 4), and the mean logMAR BCVA recovered to 0.43 ± 0.59. For BCVA at three time points, D7, D14, and D30, no significant difference was found between each other (all adjusted *p* > 0.05, Table 4).

**Table 2:** Visual acuity and OCTA parameter of the studied eyes in two group during different time points.

	Total	Control group	PRP group	*p*
BCVA (logMAR, mean + SD)				
D1				0.526
Mean + SD	0.57 ± 0.51	0.48 ± 0.42	0.67 ± 0.59	
Median (Range)	0.60 (0–2)	0.40 (0–1.22)	0.71 (0–2)	
D7				0.978
Mean + SD	0.57 ± 0.48	0.54 ± 0.41	0.59 ± 0.56	
Median (Range)	0.52 (0–2)	0.52 (0–1.3)	0.56 (0–2)	
D14				0.397
Mean + SD	0.52 ± 0.49	0.54 ± 0.39	0.49 ± 0.60	
Median (Range)	0.40 (0–2)	0.52 (0–1.3)	0.30 (0–2)	
D30				0.188
Mean + SD	0.50 ± 0.50	0.56 ± 0.41	0.43 ± 0.59	
Median (Range)	0.40 (0–2)	0.40 (0–1.3)	0.22 (0–2)	
CPD (mean + SD)				
D1				0.356
Mean + SD	36.99 ± 4.34	38.00 ± 3.94	35.97 ± 4.65	
Median (Range)	37.48 (28.22–43.70)	39.06 (31.72–43.70)	35.01 (28.22–42.86)	
D7				**0.043**
Mean + SD	36.85 ± 4.89	34.97 ± 4.59	38.73 ± 4.61	
Median (Range)	37.20 (30.89–48.74)	33.10 (30.89–45.02)	38.42 (32.11–48.74)	
D14				0.603
Mean + SD	38.02 ± 5.06	36.98 ± 4.85	39.05 ± 5.26	
Median (Range)	37.63 (30.84–50.37)	38.06 (30.84–43.50)	37.63 (32.21–50.37)	
D30				0.166
Mean + SD	40.78 ± 5.10	38.85 ± 4.89	42.71 ± 4.72	
Median (Range)	40.10 (30.16–52.15)	39.24 (30.16–47.37)	40.20 (38.01–52.15)	
Moth-eaten area index (mean + SD)				
D1				0.817
Mean + SD	0.50 ± 0.05	0.50 ± 0.04	0.50 ± 0.06	
Median (Range)	0.49 (0.42–0.61)	0.49 (0.44–0.55)	0.49 (0.42–0.61)	
D7				0.356
Mean + SD	0.46 ± 0.06	0.47 ± 0.05	0.44 ± 0.07	
Median (Range)	0.46 (0.31–0.56)	0.46 (0.42–0.56)	0.45 (0.31–0.52)	
D14				0.299
Mean + SD	0.47 ± 0.07	0.48 ± 0.05	0.46 ± 0.09	
Median (Range)	0.47 (0.30–0.59)	0.48 (0.40–0.57)	0.45 (0.30–0.59)	
D30				0.149
Mean + SD	0.47 ± 0.08	0.49 ± 0.06	0.45 ± 0.08	
Median (Range)	0.48 (0.32–0.57)	0.49 (0.40–0.57)	0.46 (0.32–0.56)	

Mann–Whitney test was used for the comparison between the two groups. NAION, nonarteritic anterior ischemic optic neuropathy; PRP, platelet rich plasma; BCVA, best-corrected visual acuity; CPD, capillary perfusion density; logMAR, logarithm of the minimum angle of resolution; SD, standard deviation; D1, 1 day before first PRP or basic treatment injection; D7, 7 days after first PRP injection or basic treatment; D14, 14 days after first PRP injection or basic treatment; D30, 30 days after first PRP injection basic treatment.Bold: *p* < 0.05.

**Table 3:** Visual acuity of the studied eyes in NAION patients during different time points.

	Control group	PRP group
*P* of BCVA	0.852	**<0.001**
*P* of CPD	0.139	**<0.001**
*P* of moth-eaten area index	0.960	0.231

NAION, nonarteritic anterior ischemic optic neuropathy; PRP, platelet rich plasma; BCVA, best-corrected visual acuity; CPD, capillary perfusion density.Bold: *P* < 0.05.

**Table 4:** The comparation of visual acuity and CPD of the studied eyes in the PRP group between two each time points.

Time point	BCVA	CPD
D1–D7	>0.999	0.683
D1–D14	0.347	0.683
D1–D30	**0.005**	**<0.001**
D7–D14	>0.999	>0.999
D7–D30	0.068	**0.027**
D14–D30	0.928	**0.027**

(Adjust *p*-value). AION, nonarteritic anterior ischemic optic neuropathy; PRP, platelet rich plasma; BCVA, best-corrected visual acuity; CPD, capillary perfusion density; logMAR, logarithm of the minimum angle of resolution; SD, standard deviation; D1, 1 day before first PRP or basic treatment injection; D7, 7 days after first PRP injection or basic treatment; D14, 14 days after first PRP injection or basic treatment; D30, 30 days after first PRP injection basic treatment.Bold: *p* < 0.05.

### Changes in OCTA parameters

Excitingly, the CPD of RPC at D7 in the PRP group was better than that in the control group (*p* = 0.043, Table 2), but no statistically significant difference was found for the remaining three time points between the two groups (*p* > 0.05, Table 2). Similarly, no significant difference was observed for the moth-eaten area index of the peripapillary SCP and DCP at four time points between the two groups (all *p* > 0.05, Table 2).

Surprisingly, when compared with the CPD value at other time points (D1, D7, D14) in sequence, a significant improvement of CPD occurred at D30 in the PRP treatment group (adjusted *p* < 0.001, *p* = 0.027, *p* = 0.027, Table 4), and the CPD values among the other three time points, D1, D7, and D14, were equivalent, ranging from 35.97 ± 4.65 to 39.05 ± 5.26 (adjusted *p* = 0.683, *p* = 0.068, *p* > 0.999, Table 4). The CPD value in the control group was equal at the four time points (*p* = 0.139, Table 3).

In contrast, the moth-eaten area index at four time points in neither the control group nor the PRP group was statistically significant (Friedman *p* = 0.960, *p* = 0.231, respectively, Table 3).

### Safety data

Among the 12 eyes that received PRP injection in our study, subconjunctival haemorrhage occurred in 10 eyes, periorbital or bulbar conjunctival bruising and swelling occurred in 5 eyes, and 3 patients felt eye distention and discomfort, but the symptoms were relieved within 1 week. Two patients had transient vision loss: one declined from 1 to 0.6, the other from 1 to 0.3, and one had a temporary IOP elevated to 46 mmHg. No other systemic adverse events occurred in the recruited subjects. At the stage of subject grouping, 1 patient in the PRP group did not receive a second PRP treatment due to personal economic problems.

## Discussion

In this study, we developed the first attempt, to our knowledge, to show that Tenon capsule injection of PRP is safe and may be capable of short-term visual acuity improvement for acute NAION patients. Our study revealed the possibility that PRP treatment may improve impaired visual function by improving the blood supply of radial peripapillary capillaries.

Studies of ocular circulation help us better understand the pathogenesis of NAION. The eyeball receives a dual vascular supply, and the ciliary artery and central retinal artery provide arterial contributions, both of which originate from the ophthalmic artery, a main branch of the internal carotid artery. The central retinal artery, an end artery, enters the optic nerve approximately 1 cm behind the eye, goes along with the optic nerve to the retina, and finally supplies the inner part of the retina (24). The outer part of the retina is separately supplied by choroidal arteries stemming from the posterior ciliary arteries (2, 8). As part of the central nervous system, the optic nerve, approximately 50 mm long from the globe to the chiasm, can be divided into four segments: the intraocular segment, intraorbital segment, intracanalicular segment, and intracranial segment. The intraocular segment can be further subdivided into three parts: prelaminar, laminar and posterior portions by the lamina cribrosa of the sclera. The laminar and prelaminar portions are supplied primarily by branches of the short posterior ciliary arteries (2, 8, 24). The posterior part receives its arterial blood from the surrounding pial plexus derived from small penetrating branches off the ophthalmic and posterior ciliary arteries (2, 8, 24). The side branches of the short posterior ciliary arteries, branches from the nearby pial arterial plexus, and choroidal vessels compose the Zinn-Haller anastomotic arterial circle, which provides arterial blood for the optic nerve head (2).

As a small-vessel disease of the anterior portion of the optic nerve, the exact cause of NAION remains unknown, and a series of vascular risk factors, such as tissue hypoxia, anemia, blood loss, relative hypotension, elevation of IOP, and thrombotic risk factors, result in acute hypoperfusion of the posterior ciliary arteries (1, 25). Systemic diseases, including systemic hypertension, diabetes mellitus, ischemic heart disease, hypercholesterolemia, nocturnal hypotension, obstructive sleep apnea, and systemic atherosclerosis, have been associated with an increased risk of NAION (1, 7, 25). In addition, individual anatomical susceptibilities, such as optic nerve drusen, papilledema, and a small, crowded optic nerve head with a small physiological cup, presumably make patients prone to worsening ischemia of the anterior optic nerve (1, 25). Following swelling of the optic nerve head, which triggers axonal and capillary compression via compartment syndrome, subsequent vasogenic and cytotoxic optic nerve edema aggravates the impairment of axonal flow and capillary perfusion of the optic nerve head with subsequent axonal degeneration and loss of retinal ganglion cells through apoptosis (1). As these cells die off, they are unable to create new connections with other neurons, leading to further deterioration of vision over time.

As a tissue regeneration technique, PRP treatment can delay aging in the presence of cytokines and growth factors and promote the production of type 1 procollagen and hyaluronic acid, which in turn improves the skin texture, firmness, and color homogeneity and exhibits efficacy in facial plastic surgery (12). In ophthalmology, it has been employed for ocular surface (14–17, 26–30) and as adjuvant therapy in pathogenic myopic retinal holes or idiopathic large macular holes (21, 22), showing encouraging results in the improvements of visual field (VF), multifocal electroretinography (mfERG), and microperimetry (MP) in retinitis pigmentosa (RP) patients (31).

Benefiting from the emergence of OCTA, which provides detailed images of retinal and choroidal blood flow, allows us to noninvasively visualize microvascular flow impairment. Chen found decreased retinal nerve fibre layer (RNFL) microcirculation in eyes with open-angle glaucoma and in the normal hemisphere of eyes with glaucoma, as measured by blood flux index and vessel area density, and both were significantly correlated with visual field loss and RNFL thinning, which suggests that vascular dysfunction may precede structural changes in glaucoma and that microcirculation measurement may help physicians monitor glaucoma (23, 32). Similarly, as an optic neuropathy, poor blood flow recovery of the optic disc coincided with the poor visual function prognosis in nonacute phase NAION (33). Wright Mayes discovered that both the RPC and peripapillary choriocapillaris (PCC) were affected in patients with NAION (34). Due to the decrease in accuracy of PCC caused by papilledema and the decrease in visual field credibility caused by low vision, PCC and visual field indicators were not included in our study for analysis.

In our study, we exhibited an improvement in radial peripapillary capillaries after PRP injection, as shown by a significant improvement in CPD observed at D30 in the PRP group (adjusted *p* < 0.001, *p* = 0.027, *p* = 0.027, compared with D1, D7, D14, Table 4); correspondingly, a better BCVA occurred at D30 compared with BCVA at D1 (adjusted *p* = 0.005, Table 4). Although no significant change was found at D7 or D14 (adjusted *p* > 0.999, *p* = 0.347, respectively, Table 4), ocular adverse effects cannot be ignored within the first week after PRP injection, which may affect the assessment of visual acuity. Interestingly, a better CPD occurred at D7 in the PRP group than in the control group (*p* = 0.043, Table 2); regrettably, no statistically significant difference was found for the BCVA between the two groups (all *p* > 0.05, Table 2). The possible reason may be that the improvement in visual function within the group was not sufficient to achieve a group difference. Furthermore, a smaller sample size, 12 for the PRP group and 13 for the control group, may also cause a bias in our results.

Although an increasing number of studies have reported that PRP injection is a safe, virtually pain-free, simple, and rapid treatment (14), with no risk of infection and transmission of disease, minor adverse effects such as discomfort, small prick bleeding, and swelling can occasionally occur (35), and a rare but devastating complication—irreversible visual loss due to occlusion or impairment of the central retinal artery and the posterior ciliary artery secondary to PRP injection—should not be overlooked (23, 36–38).

Our study surely has several limitations. First, only twelve patients were included in the PRP group with a wide range of unequal logMAR VA before PRP injection from 0 to 2, which may cause a bias in our results. The number of patients in the control group was also relatively small, and an expanded sample size study is still needed to verify our conclusions. Second, as a human-based experimental study, although two groups with equivalent baselines were included in our study, we assigned patients largely dependent on personal preference, and it is difficult to perform a random double-blind test. An interaction effect between basic treatment and PRP injection is unavoidable. Third, due to the impact of the novel coronavirus, we did not complete a continuous follow-up, and it is essential to assess the long-term effect of PRP injection for NAION patients. Last, because of the poor reliability of visual field assessments, we only chose visual acuity as a single functional indicator and OCTA parameters as the unique structural indicator. A further comprehensive assessment is indispensable to provide more information about whether PRP treatment could improve visual acuity by improving blood perfusion of the optic nerve.

The most prominent points of this study are providing an opening for NAION’s new treatment, Tenon capsule injection of PRP, which can significantly improve capillary perfusion of the optic nerve head and might be helpful in increasing short-term vision in patients with acute NAION.

## Data availability statement

The original contributions presented in the study are included in the article/supplementary materials, further inquiries can be directed to the corresponding authors.

## Ethics statement

The studies involving humans were approved by The Third Medical Center of the Chinese People’s Liberation Army General Hospital is affiliated with the Third Medical Center of the Chinese People’s Liberation Army General Hospital. The studies were conducted in accordance with the local legislation and institutional requirements. The participants provided their written informed consent to participate in this study.

## Author contributions

XJ: Conceptualization, Investigation, Writing – review & editing, Project administration, Supervision. JF: Writing – original draft, Formal analysis, Validation. SW: Data curation, Investigation, Writing – review & editing. RL: Investigation, Writing – original draft. XH: Investigation, Writing – original draft. MS: Investigation, Writing – original draft. GX: Investigation, Writing – original draft. QZ: Investigation, Writing – original draft. LZ: Resources, Writing – original draft. YL: Resources, Writing – original draft. QX: Project administration, Writing – original draft. BH: Formal analysis, Investigation, Visualization, Project administration, Writing – original draft.
